# Systematic Monitoring of Cognition for Adults With Cerebral Palsy—The Rationale Behind the Development of the CP*Cog*-Adult Follow-Up Protocol

**DOI:** 10.3389/fneur.2021.710440

**Published:** 2021-09-22

**Authors:** Kristine Stadskleiv, Marleen R. van Walsem, Guro L. Andersen, Lena Bergqvist, Louise Bøttcher, Klaus Christensen, David Heyerdahl, Sandra Julsen Hollung, Helene Høye, Reidun Jahnsen, Gunvor L. Klevberg, Barbro Lindquist, Henrik Passmark, Per-Ola Rike, Elisabet Rodby-Bousquet, Ann I. Alriksson-Schmidt

**Affiliations:** ^1^Department of Special Needs Education, University of Oslo, Oslo, Norway; ^2^Department of Clinical Neurosciences for Children, Oslo University Hospital, Oslo, Norway; ^3^Department of Neurohabilitation, Oslo University Hospital, Oslo, Norway; ^4^Center for Habilitation and Rehabilitation Models and Services, Institute of Health and Society, University of Oslo, Oslo, Norway; ^5^Norwegian Quality and Surveillance Registry for Cerebral Palsy (NorCP), Vestfold Hospital Trust, Tønsberg, Norway; ^6^Unit of Occupational Therapy, Department of Health and Rehabilitation, Institute of Neuroscience and Physiology, Sahlgrenska Academy, University of Gothenburg, Gothenburg, Sweden; ^7^Danish School of Education, Aarhus University, Copenhagen, Denmark; ^8^CP Denmark, Taastrup, Denmark; ^9^The Norwegian Cerebral Palsy Association, Oslo, Norway; ^10^Varden Specialist Center, Bjørnemyr, Norway; ^11^Norwegian Quality and Surveillance Registry for Cerebral Palsy (NorCP), Department of Clinical Neurosciences for Children, Oslo University Hospital, Oslo, Norway; ^12^Habiliation Center, Halmstad, Sweden; ^13^The Cerebral Palsy Surveillance Programme (CPUP), User board, Lund, Sweden; ^14^Department of Research, Sunnaas Rehabilitation Hospital, Nesoddtangen, Norway; ^15^Center for Clinical Research, Uppsala University-Region Västmanland, Västerås, Sweden; ^16^Department of Clinical Sciences Lund, Orthopaedics, Lund University, Lund, Sweden

**Keywords:** cerebral palsy, cognition, assessment, intelligence, transitioning, life-span, health service access

## Abstract

Cerebral palsy (CP) comprises a heterogeneous group of conditions recognized by disturbances of movement and posture and is caused by a non-progressive injury to the developing brain. Birth prevalence of CP is about 2–2.5 per 1,000 live births. Although the motor impairment is the hallmark of the diagnosis, individuals with CP often have other impairments, including cognitive ones. Cognitive impairments may affect communication, education, vocational opportunities, participation, and mental health. For many years, CP has been considered a “childhood disability,” but the challenges continue through the life course, and health issues may worsen and new challenges may arise with age. This is particularly true for cognitive impairments, which may become more pronounced as the demands of life increase. For individuals with CP, there is no one-to-one correlation between cognition and functioning in other areas, and therefore, cognition must be individually assessed to determine what targeted interventions might be beneficial. To facilitate this for children with CP, a systematic follow-up protocol of cognition, the CP*Cog*, has been implemented in Norway and Sweden. However, no such protocol currently exists for adults with CP. Such discontinuity in healthcare services that results from lack of follow-up of cognitive functioning and subsequent needs for adjustments and interventions makes transition from pediatric to adult healthcare services challenging. As a result, a protocol for the surveillance of cognition in adults with CP, the CP*Cog*-Adult, has been developed. It includes assessment of verbal skills, non-verbal reasoning, visual–spatial perception, and executive functioning. It is recommended to perform these assessments at least once in young adulthood and once in the mid-fifties. This report describes the process of developing the CP*Cog-*Adult, which has a three-fold purpose: (1) to provide equal access to healthcare services to enable the detection of cognitive impairments; (2) to provide interventions that increase educational and vocational participation, enhance quality of life, and prevent secondary impairments; and (3) to collect systematic data for research purposes. The consent-based registration of data in the well-established Swedish and Norwegian national CP registries will secure longitudinal data from childhood into adulthood.

## Introduction

Cerebral palsy (CP) is a condition that comprises a heterogeneous group of individuals with motor impairments due to a congenital brain malformation or an early acquired brain lesion. The motor disability is often accompanied by other impairments, of which cognitive functioning is one of the areas frequently affected (1). Cognitive impairments can have far-reaching consequences, and affect academic learning, social functioning, self-care skills, and participation in society. There is a large variability in the type and severity of cognitive impairments among individuals with CP, ranging from challenges affecting functioning in just one area to severe and global impairments (2, 3). Although cognition and motor functioning are associated, there is not a one-to-one relationship (4) and individual assessment of cognition is therefore recommended (5).

CP has for many years been regarded as a “childhood disability” in the sense that interventions have been mainly targeting children and adolescents. To ensure that all children with CP are offered individual assessments of cognition, a protocol of cognition for children with CP (the CP*Cog*) was developed in Scandinavian countries (i.e., Sweden, Denmark, and Norway) (6). Research emerging over the last decades has shown, however, that challenges continue into adulthood, when pre-existing problems worsen and new challenges may arise (7, 8). Despite this, there is no systematic follow-up of cognition in adults with CP in Scandinavian countries. In addition to challenges experienced by most young adults, such as gaining independence, completing an education or a training, gaining employment, and starting a family, adults with CP also report specific challenges, including increased experiences of pain, mental fatigue, and loss of functioning. Thus, although the initial brain lesion is non-progressive, the consequences may change over time (9–18). This is also true for cognitive impairments (11). Specific cognitive impairments, such as executive dysfunctioning (19) and visual–perceptual impairments (20), play a larger role as expectations of academic functioning and independence increase. Adults with CP are less likely to be employed than their adult counterparts without CP, even after completing regular education and having normal intelligence (21). Sometimes, the reason is lack of physically accessible workplaces or sufficient adaptation to perceptual problems, but mental fatigue, pain, and lack of compensatory aids may also contribute. Being excluded from employment has ramifications other than the obvious financial ones, as work not only is a source of income, but also provides companionship, purpose, and feelings of being able to contribute and participate (22). For society, it is preferable that people who are capable of employment are working, and necessary adaptations should therefore be put in place to enable employment for as many as possible (23). Moreover, in 2006, the United Nations published the *Convention on the Rights of Persons with Disabilities* (CRPD), which mandates in-home, residential, and community support services, including personal assistance necessary to prevent isolation and segregation by supporting living and inclusion in the community (24). Scandinavian countries have all ratified the CRPD (25).

Scandinavian countries have public health and welfare systems that are available for all at low costs. In Sweden and Norway, services to children and adults with congenital and early acquired brain injuries are offered through the habilitation centers, while adults who acquire brain injuries are offered services through rehabilitation hospitals and centers (26). The habilitation centers for adults are a part of the specialized healthcare services, and as each serves a limited geographical cohort of inhabitants, together they provide national coverage in each country. In Sweden, there are a total of 137 habilitation centers for adults throughout the 21 hospital regions (27). In Norway, there are 20 hospital trusts that offer habilitation services to adults, with a total of 31 teams/locations (28). Denmark does not have a system of habilitation centers, neither for children nor for adults, but follow up individuals with disabilities in the municipalities.

The first systematic Scandinavian surveillance program for CP was established in Sweden in 1994, and during the next 16 years, it spread to Norway and Denmark (29). The Swedish Cerebral Palsy Follow-up Program (CPUP), the Norwegian Quality and Surveillance Registry for Cerebral Palsy (NorCP), and the Danish Cerebral Palsy Follow-up Program (CPOP) are combined quality registries and surveillance programs (30, 31) [The NorCP in Norway includes the former Cerebral Palsy follow-up program (CPOP) and the Cerebral Palsy Registry of Norway (CPRN), which were combined in 2020 (32)]. The surveillance programs have primarily focused on motor functioning. The cognitive follow-up protocol for children, the CP*Cog*, was first introduced in Norway in 2012 and in Sweden in 2015 (33). In Denmark, the need for cognitive follow-up of children with CP has been recognized, but implementation of CP*Cog* has been challenging partly due to the organization of the healthcare services, which lack a similar structure to Swedish and Norwegian habilitation centers. In Norway, the introduction of the CP*Cog* has led to more children with CP being offered assessments of cognition. Before the introduction, only 29% of children with CP had been assessed with a standardized test of intelligence (30). After increasing knowledge about the CP*Cog* protocol in the pediatric habilitation centers through a quality improvement project in 2019, this number increased to 54% (34). In Sweden, over the last 5 years, 14 of the 21 regions have started to record cognitive data in the CPUP database, and by December 2020, 292 children (~7% of all children with CP in Sweden) had undergone cognitive assessments [unpublished results].

For persons with disabilities, it is vital to have proper knowledge and understanding about personal strengths and challenges in order to be able to describe the type of adaptations that are required (10, 35). These adaptations pertain not only to physical accessibility, but also to adaptations necessary to handle the cognitive demands of education, work, or of meaningful and fulfilling activities if employment is not an option. For instance, the appropriate type of communication aid is central for adults with CP who have speech impairments (17, 23, 36). This implies that adults with CP need to have detailed knowledge about their cognitive functioning and needs.

Despite the vast impact cognitive impairments might have on the possibilities of independent living, employment, communication, and general wellbeing among adults with CP, very few studies have focused on cognitive functioning or on interventions aimed at increasing the management of cognitive impairments (37–39). The only exceptions being some studies that highlight the executive difficulties adults with dyskinetic CP might experience (40, 41). In addition, there are very few longitudinal studies following cognition in cohorts of children with CP (2), and as far as we are aware, there is only one longitudinal study that includes adults (8). In this study, however, cognition was not assessed, but was included as one of several dimensions in a questionnaire on quality of life on which the adults reported functioning.

Given the identified clinical needs and the lack of studies of cognitive functioning in adults with CP, the decision was made to extend the current systematic surveillance program for cognition in children and youths, the CP*Cog*, to adults with CP. The aim of this article is to describe the rationale behind the protocol for the systematic assessment of cognition in adults with CP—henceforth referred to as the CP*Cog*-Adult—and how this protocol may enhance access to healthcare services in Norway and Sweden.

## Materials and Equipment

### Participants

All persons 18 years or older living in Norway and Sweden with a diagnosis of CP (42) are eligible for inclusion. The prevalence of CP is now ~2–2.5 per 1,000 live births in Scandinavian countries, which implies that, per birth year, there is ~140–165 living with CP in Norway and Denmark and 200–220 in Sweden (31, 43–45). However, for adults, the prevalence is probably lower. Based on Swedish registry data, it was recently reported to be as low as 1.1 per 1,000 (46).

### Instruments

The core battery of instruments chosen to assess cognitive functioning was selected based on the following criteria: (1) equivalence to instruments included in the pediatric surveillance protocol CP*Cog*, (2) their current use and availability in the habilitation centers, and (3) availability of national or Scandinavian norms. The core battery therefore consists of a standardized test of intelligence, the Wechsler Adult Intelligence Scale (47, 48), a test of visual–spatial abilities, the Beery-Buktenica Developmental Test of Visual-Motor Integration (49), and the questionnaire Behavior Rating Inventory of Executive Function (50, 51) for self- and proxy report on executive functioning in daily life (see Table 1).

**Table 1:** Instruments in the CP*Cog*-Adult for assessing cognition and adaptive functioning: core battery (recommended for all) and supplemental tests (administered on indication).

**Area**	**Test**	**Task**	**Type of instrument**	**Included in**
**Cognition**	Wechsler Adult Intelligence Scale (47, 48)	At minimum tasks necessary for a IQ score	Test	Core battery
	The Beery-Buktenica Developmental test of Visual-Motor Integration (49)	Copy, visual perception, and motor integration	Test	Core battery
**Attention and executive functioning**	Behavior Rating Inventory of Executive Function—Adult (50, 51)	Self-report and informant report	Questionnaire	Core battery
	Wisconsin Card Sorting Test (52)		Test	Supplemental battery
	Delis-Kaplan Executive Function System (D-KEFS) (53, 54)	Trail Making Test and the Color–Word Interference	Test	Supplemental battery
**Memory**	California Verbal Learning Test (55)		Test	Supplemental battery
	Rey Complex Figure Test (56)		Test	Supplemental battery
**Adaptive functioning**	Vineland Adaptive Behavior Scales (57, 58)	Informant report	Informant interview/questionnaire	Supplemental battery
**Fatigue**	Fatigue Severity Scale (59)		Questionnaire	Supplemental battery^*^
	Modified Mental Fatigue Scale (60)		Questionnaire	Supplemental battery
**Pain**	Short Form Health Survey-36 (SF-36) (61)	Section about pain	Questionnaire	Supplemental battery^*^
	World Health Organization Disability Assessment Schedule (WHODAS 2.0) (62)	The 12-item version	Questionnaire	Supplemental battery^*^
**Mental health**	Hospital Anxiety and Depression Scale (63)		Questionnaire	Supplemental battery

* *Already included in the follow-up program (CPUP) for adults with CP in Sweden*.

In addition to the core instruments in CP*Cog*-Adult, it is recommended to use instruments for more thorough assessments of attention, executive functioning, and memory, if challenges in these areas are detected. For this purpose, tasks from the Delis-Kaplan Executive Function System (53, 54), such as the Trail Making Test and the Color–Word Interference, the California Verbal Learning Test (55), the Rey Complex Figure Test (56), and the Wisconsin Card Sorting Test (52) are recommended. If participants report, or mental health issues are noted, it is recommended to administer the Hospital Anxiety and Depression Scale (63), and that referrals are provided to receive professional help, as appropriate. If a diagnosis of intellectual impairment is suspected, it is recommended to use instruments such as the Vineland Adaptive Behavior Scale (57, 58).

Cognitive assessments of individuals with the most severe motor impairment include adaptations, such as substituting finger pointing with gaze pointing on a computer screen using tests with a multiple-choice format (64). It is therefore recommended that cognitive assessments of adults with the most severe speech and motor impairments are offered at habilitation centers with specialized knowledge about the use of assistive technology and augmentative and alternative communication (AAC) and that funding is applied to develop this knowledge when necessary.

In Norway, it is recommended to use instruments for assessment of fatigue, pain, self-care abilities and activities of daily living, as this might affect participation in everyday life and employment. Suggested instruments are the Fatigue Severity Scale (FSS) (59) or the Modified Mental Fatigue Scale, which is developed particularly for adults with CP (60), the section on pain from the Short Form Health Survey-36 (SF-36) (61), and the 12-item version of the World Health Organizations Disability Assessment Schedule 2.0 (WHODAS 2.0) (62). The FSS, WHODAS 2.0, and pain questions from SF-36 are already part of CPUP for adults in Sweden.

### Assessment Time Points

The CP*Cog*-Adult includes two recommended assessment points, and two additional time points, which are proposed to be optional (see Table 2). This assessment schedule will enable long-term follow-up of the participants, as well as longitudinal studies.

**Table 2:** Recommended time points for the assessment of cognition and adaptive functioning in adults with cerebral palsy.

**Age**	**Rationale**	**Status**
18–19 years	Knowledge of cognitive functioning may aid in planning education and housing. Recommend assessment if not done during adolescence.	Optional
24 years	Aid in transitioning from education to employment. First time during adulthood that all with CP are scheduled for follow-up as part of CPUP-Adult in Sweden.	Highly recommended
42 years	Approximately midway through employment. Highly recommended for adults with bilateral CP, to prevent late adverse effects with respect to pain and fatigue, which might be particularly prevalent in this group.	Optional
54 years	Knowledge of cognitive functioning may be beneficial to prevent early retirement or transition to disability benefits due to health reasons.	Highly recommended

These time points are guidelines and might need to be adjusted somewhat for feasibility. Furthermore, if an adult has recently (i.e., within the last 5 years) been cognitively assessed as part of their clinical follow-up, assessment should not be repeated for the mere sake of following the CP*Cog*-Adult protocol. For example, if a 24-year-old adult has already been assessed sometime between 19 and 23 years of age, he/she should not be reassessed at 24 years of age unless it is deemed appropriate for another reason.

## Methods

The initiative to develop the CP*Cog* protocol for children was taken by the umbrella organization for the user organizations in the Nordic countries (CP Norden) and the subsequent development was described as the result of a natural experiment (6). Based on our experiences from this (34) and the development of procedures to systematically follow-up adults with CP in other domains (65, 66), professionals in Scandinavia involved in the development of the CP*Cog* took the initiative to develop the CP*Cog-*Adult (In 2012, an initiative was taken to include the other two Nordic countries, Iceland and Finland, in the development of the CP*Cog* protocol, but was unfortunately not successful at that time).

The development process started in the spring of 2019. Initially, meetings and discussions were country-specific. Next, professionals from Scandinavian countries met at Lund University, Sweden, in April 2019, after which a first draft of the protocol was sent out to professionals and user representatives in all three Scandinavian countries. This draft was then discussed in a meeting consisting of representatives, both professionals and users, from Scandinavian countries at the CPUP conference in Stockholm, Sweden, in October 2019. Further amendments of the protocol were discussed *via* email, until consensus was reached. The proposed protocol is therefore the result of discussions and joint collaboration between users, clinicians, and researchers (i.e., the authors of this manuscript).

## Anticipated Results

The protocol has a threefold purpose: (1) to provide equal access to healthcare services that will enable the detection of cognitive impairments; (2) to provide interventions that increase educational and vocational participation, enhance quality of life, and prevent secondary impairments; and (3) to collect systematic data for research purposes.

### Access to Healthcare Services

The sample has the potential to constitute large geographical cohorts in the two countries. The habilitation centers for adults are part of the public health services, and as such, the costs for the individuals using them are minimal. This ensures that the CP*Cog*-Adult protocol will be accessible to all adults with CP, regardless of their socio-economic background, once the protocol is implemented.

It is recommended that the habilitation centers for adults take the primary responsibility of recruiting participants, as they are the part of the public healthcare systems who sees the largest number of adults with CP. This, however, depends on the habilitation centers' resources and willingness to implement the protocol. We have reason to believe that many centers will regard this initiative positively, given that all pediatric habilitation centers in Norway volunteered to be part of the quality improvement project in 2019, aimed at implementing the CP*Cog*-protocol for children (34). Also, the vast majority of healthcare regions in Sweden participate in the protocol for children.

Recruitment might initially be somewhat challenging, since many adults with CP are currently not followed regularly by the adult habilitation centers. These individuals are not always known to the specialized healthcare system, and will therefore not receive invitations for cognitive assessments. Instead, they will be approached and enrolled on a consecutive basis in the follow-up protocol as soon as they get in contact with the healthcare system. This challenge is expected to particularly affect assessments for individuals at 54 years of age, and to be less of concern at 24 years of age. The reason for this is that the younger adults have been enrolled in the follow-up programs for children that cover over 95% of children with CP. When transitioning into adult services, the habilitation centers for children can inform participants about the possibility of continued follow-up in adulthood. As such, the introduction of the CP*Cog*-Adult protocol will secure the continuity of clinical care when transitioning from pediatric to adult services. Over time, the aim is therefore that the protocol will be offered to all adults with CP.

### Enabling Interventions

The rationale for the areas investigated and the recommended assessment time points in the CP*Cog*-Adult protocol are related to securing appropriate interventions with regard to education and employment. Due to lack of longitudinal studies, it was not possible to base recommendations on developmental trajectories of cognitive functioning in adults with CP. However, the suggested time points are harmonized with the existing follow-up protocol for adults with CP in Sweden (CPUP for adults). The time points are, however, only guidelines and data for individuals who are assessed at other ages can still be entered into the national databases, provided consent.

Around 18–19 years of age, there is a transition from the school system where everybody is included and everyday life is structured, into a more uncertain, less structured future that requires more active choices by the individual. Many go on to higher education, some start work, either in regular or in sheltered employment, others are enrolled in day service centers, whereas some do not gain access to further education or employment. The education, employment, and everyday activities people are offered should reflect their cognitive, communicative, and social skills, and not be limited by lack of knowledge about each individual's potential. However, many of the younger adults with CP will have been included in the follow-up programs for children, where an assessment of cognition is recommended at 15 years of age as an optional part of the CP*Cog* (6). This time point is only considered necessary for the young adults who have not been assessed as teens and is therefore listed as optional.

The first age at which all adults with CP should be offered cognitive assessment is around 24 years of age. At this age, those who have been enrolled in higher education usually transition to employment. However, even for those who have successfully completed higher education, transitioning to employment can still be challenging (21). Subtle cognitive deficits that may not have been as noticeable when living at home and attending school may have serious consequences when transitioning to employment and being expected to manage independent living. Offering a cognitive assessment at this age, and thereby detecting subtle deficits (for example, in the ability to initiate, plan, organize, and juggle a number of tasks at the same time), will make it possible to put in place the necessary adjustments in the workplace and at home. Moreover, 24 years of age fits with the continuous follow-up in other areas offered to adults within the CPUP program in Sweden. In Sweden, the follow-up of adults is scheduled annually, every second year, or every third year based on their functional level according to the Gross Motor Function Classification System (67). Therefore, 24 years of age will be the first time point after turning 18 years when all adults with CP will be seen by the habilitation centers in Sweden.

Another optional assessment time point is suggested at 42 years of age. At this age, many are approximately halfway through their working years. The purpose of having an assessment at this time point is to prevent adverse late effects with respect to pain and fatigue, which might be particularly relevant for adults with bilateral CP (15).

At 54 years of age, the last quartile of work-life is approaching. Many with chronic disabilities, who have managed to stay employed over the years, are forced into early retirement or to transition to disability benefits due to health reasons (9, 68). It is therefore suggested that all adults with CP are offered an assessment of cognition in their mid-fifties, and 54 years of age is recommended, as this is an age when all will be seen as part of the CPUP for adults. The purpose of this is to implement, if necessary, interventions that will enable adults with CP to stay connected to the workforce as long as possible or to transition gradually to early retirement.

### Data Registry

The CP*Cog*-Adult protocol aims to ensure that all adults with CP in Norway and Sweden are offered cognitive assessments around 24 and 54 years of age, at a minimum. Inclusion into the CP*Cog*-Adult will be based on participants' informed consent, according to national standards. With consent, data from their clinical cognitive assessment can be entered into the national CP registries. It may be valuable to register any adaptations to testing procedures that are made, as this will aid in interpreting the results and provide unique epidemiological data on assessment procedures. It will be possible to consent to only participation in the cognitive assessment, and not to registration of data, but experience from the pediatric protocols indicates that the vast majority consent to registration of data (31).

Moreover, it will be possible to merge data from assessments in childhood with assessment in adulthood. Therefore, over time, the national CP registries will provide unique longitudinal data on the developmental trajectories of individuals with CP.

## Discussion

The CP*Cog*-Adult protocol has a threefold purpose: (1) to allow for systematic clinical follow-up of cognition in adults with CP, (2) to provide equal access to health services, and (3) to strengthen research on CP in adulthood.

The primary goal of the CP*Cog*-Adult protocol is to increase the quality of life of adults with CP, i.e., that their strengths and challenges will be recognized, interventions that may prevent dropout from education and employment may be implemented, and the development of secondary impairments resulting from unrecognized cognitive challenges can be prevented. Failing to gain employment and falling out of the workforce can lead to social isolation, depression, and secondary somatic problems (22). Cognitive functioning is not only important for learning to live with and managing challenges related to daily life both at home and work, but awareness of cognitive impairments may also contribute in the prevention of fatigue, mental distress, and reduced quality of life, by allowing necessary interventions to be implemented.

Studies of cognitive functioning in children with CP indicate that not all children keep their level of cognitive functioning over time compared to their peers who do not have CP (69, 70). A larger proportion of youths with CP have intelligence quotient (IQ) scores in the low range, compared to children in the younger age cohorts. This cannot be explained by worsening of motor functioning, as the same finding is also seen for tests placing very low demands on motor functioning (5). Even if children with CP improve their skills, the gap between their level of functioning and that of their peers without disability may increase (2). This illustrates the necessity to assess cognition not only in childhood, but also in adulthood.

Transitioning from child to adult healthcare services is a vulnerable time, and for the transition to be successful, it must be well-planned (71). All children with CP are followed by the local pediatric habilitation centers until they are between 16 and 18 years of age, and thereafter, they transition into adult services. Some of the older participants in the CP follow-up programs/registries, born between 1994 and 2001, have now reached adulthood and may be expecting the same systematic follow-up as they received as children and adolescents. However, this is currently not guaranteed for all geographical areas. In Sweden, adults with CP are followed by the CPUP program, but this program does not focus on cognition. In Norway, adults with CP who have an intellectual disability or recognized cognitive deficits are traditionally those most likely to be followed by the adult habilitation centers. In addition, they will also often be offered community services like housing and sheltered work. The rest of the adult CP population is usually followed by a family physician or a primary healthcare clinic. Healthcare for adults tends to be less multidisciplinary and more fragmented and requires more personal responsibility in terms of arranging and getting to appointments and coordinating different types of services. Many adults with CP or other disabilities thus report that they feel left to cope on their own (65, 72).

An additional reason to develop a follow-up protocol for cognitive monitoring is therefore to facilitate equal access to health services, which focus on cognition and the possible consequences of cognitive impairments for all adults with CP. For this purpose, the follow-up protocol only has value if it is widely implemented. The results from the CP*Cog* for children illuminate that offering assessments in a systematic manner to all increases the numbers reached, but it also illustrates that changing clinical practice takes time. Comprehensive projects in other countries have shown that managing a successful transition from child to adult health services requires an organization-wide approach to implementation (71). Introducing the CP*Cog*-Adult protocol, which builds on the CP*Cog* protocol for children, is an important part of such a multidisciplinary intervention approach. However, implementation of the CP*Cog*-Adult will depend on securing support for the protocol among relevant stakeholders, including the managers in charge of the adult habilitation centers and the psychologists. Therefore, the proposed protocol is comprehensive enough to yield meaningful results, while at the same time not requiring more resources than deemed absolutely necessary. It is difficult to estimate the time needed for assessments, due to the large heterogeneity of the CP population. However, the majority of assessments are expected to be completed in two sessions lasting from 1 to 2 h each time.

In Norway and Sweden, the adult habilitation centers already provide services to some adults with CP. In Sweden, there are regions where all adults with CP are offered follow-up as part of the CPUP, and where transitioning from pediatric to adult services therefore is already handled well. In Norway, follow-up by the adult habilitation centers, which are run by 20 hospitals spread throughout the country (28), is typically dependent on referral and not part of systematic follow-up of the whole cohort (66). In Norway, to secure the referral of adults with CP to the adult habilitation centers, the pediatric habilitation centers will be encouraged to inform adolescents about the protocol when they turn 18 years of age. Professionals at other rehabilitation institutions that provide more specific and time-limited rehabilitation services to adults with CP will also be encouraged to refer, and the protocol will be made known to adults with CP through the user organization's journal, webpage, and annual user conference. Currently, the centers are not staffed to implement a systematic cognition follow-up protocol for adults with CP; thus, additional funding needs to be secured (73). Offering assessments to all adults with CP at two time points implies that, for Norway with ~80–90 adults with CP per birth year (46), 160–180 assessments are to be conducted nationally per year (43), which, in turn, implies an average of 8–9 assessments annually per hospital. This does not imply that there will be a need for 180 assessments in addition to what the centers are already conducting, as many of the adults with CP will already have been scheduled for assessment or have been assessed within the last 5 years. Furthermore, the adult habilitation centers may cooperate with other services offering neuropsychological assessments of adults with CP, like the Sunnaas Rehabilitation Hospital in Norway. However, it is still likely that implementation of the CP*Cog*-Adult protocol will lead to an increase in the number of assessments offered, thus indicating a need for increased resources allocated to the adult habilitation centers.

The primary aim of the CP*Cog*-Adult is clinical; to detect challenges in the assessed areas so that more in-depth assessments, interventions, or referrals might be undertaken as needed. However, it is also an aim to create a better knowledge base for research related to cognitive functioning in adults with CP. On a consent basis, information from the individual assessments, coupled with information about diagnosis, functioning, type of brain lesions, and associated impairments, will be entered into the national CP registries (CPUP and NorCP). The national CP registries can be merged with other national health registries, pending ethical approval. A Nordic research project based on the Nordic CP registries and other national registries, “CP-North—Living life with cerebral palsy in the Nordic countries?,” is ongoing, funded by Nordforsk (https://rdi.arcada.fi/cpnorth). There is also an ongoing research program, MOVING ON with CP, which is funded by the Swedish Research Council for Health, Working Life and Welfare, where the development and implementation of CP*Cog* and CP*Cog*-Adult are included (74). As multiple assessments spanning across many years are planned, longitudinal studies on cognitive functioning from childhood into adulthood will also be possible. Studies revealing more comprising descriptions of cognitive function in light of other clinical and socio-demographic data of the adult population with CP at various points across adult life will be conducted. Linking the clinical follow-up protocol CP*Cog*-Adult with the national CP registries will therefore be beneficial for research, which in turn will lead to quality improvement of services.

### Strength and Limitations

The initiative to establish the CP*Cog* for children came as a joint initiative from the user organizations in the Nordic countries, illustrating that families recognize that cognition is a concern that has traditionally been overlooked. A strength to this initiative is that the CP*Cog*-Adult protocol has been discussed and finalized together with representatives from the user organizations, in collaboration with representatives from the national CP registries and follow-up programs (CPUP and NorCP) and the specialized health services in Sweden and Norway.

The CP*Cog*-Adult protocol outlines a systematic manner of assessment, detailing both assessment time points and instruments to be used. A limiting factor is that the protocol only details assessment, not the interventions that should follow from the results or a plan for psychoeducational follow-up. The aim is to, over time, expand the protocol, so that it, in the future, also includes suggestions for interventions. An additional limitation is that the protocol likely will be implemented at different time points in Sweden and Norway and that funding is not yet fully secured. It is a strength that the battery of neuropsychological tests also includes tests that can be administered to adults with severe motor impairments and that cognition is assessed together with adaptive skills in adults where a diagnosis of adaptive functioning is possible. Furthermore, it is a limitation that only two time points are recommended and that the interval between these (i.e., 24 and 54 years of age) is large. The rationale for recommending only two time points is to propose a protocol with a limited enough scope to make it feasible for implementation. The drawbacks of this approach are that each adult with CP will been seen by a psychologist very seldom, that the follow-up might end too early to detect any neurodegenerative changes that adults with brain lesions are potentially more at risk for, and that it will take a long time to collect longitudinal data. A final potential limitation is that the number of instruments suggested is rather large, especially if administering both the core and the supplemental battery. It is therefore necessary to investigate the feasibility of the protocol in a real-life clinical situation, and the plan is therefore to apply for funding for a pilot project to investigate this further.

### Clinical Implications

Adults with CP have an increased risk of cognitive impairments, which are not always recognized but may have negative consequences. A systematic follow-up of cognition in adults with CP is therefore needed. This need for follow-up led to the development of the CP*Cog*-Adult protocol, which comprises a battery of instruments to assess cognition, and will be offered to all adults with CP at specified time points (around 24 and 54 years of age). This protocol is brief by design, and only two time points are highly recommended while a further two time points are listed as optional. This is done to maximize the likelihood that the protocol will be implemented. Representatives from user organizations have collaborated with clinicians and researchers to develop the protocol, and will collaborate also in disseminating experiences at workshops and training courses for relevant professionals, at scientific conferences, in written publications, on the websites of the user organizations, and in relevant scientific journals. Implementation of the CP*Cog-*Adult protocol will be carried out in close collaboration with the leaders of the adult habilitation centers in Norway and Sweden.

In addition to securing better follow-up of cognition in adults with CP, the protocol is also designed to increase our knowledge about cognitive impairments and the consequences, which may follow. To enable the best possible data collection in Scandinavian countries, the protocol builds on the CP*Cog* protocol for children, and will be harmonized with follow-up in other areas of adults with CP.

## Data Availability Statement

The original contributions presented in the study are included in the article/supplementary material, further inquiries can be directed to the corresponding author/s.

## Author Contributions

All authors listed have made a substantial, direct and intellectual contribution to the work and approved it for publication.

## Funding

This project was financially supported by Forte (# 2018-01468). The findings and conclusions in this report are those of the authors and do not necessarily represent the official position of the funders. The Cerebral Palsy Follow-up Program in Sweden (CPUP) covered the authors' traveling costs for participation in a meeting in Lund, on April 12, 2019.

## Conflict of Interest

The authors declare that the research was conducted in the absence of any commercial or financial relationships that could be construed as a potential conflict of interest.

## Publisher's Note

All claims expressed in this article are solely those of the authors and do not necessarily represent those of their affiliated organizations, or those of the publisher, the editors and the reviewers. Any product that may be evaluated in this article, or claim that may be made by its manufacturer, is not guaranteed or endorsed by the publisher.
